# Small Bugs, Big Data: Metagenomics for Arthropod Biodiversity Monitoring

**DOI:** 10.1002/ece3.72163

**Published:** 2025-09-16

**Authors:** Samantha López Clinton, Ela Iwaszkiewicz‐Eggebrecht, Andreia Miraldo, Robert Goodsell, Matthew T. Webster, Fredrik Ronquist, Tom van der Valk

**Affiliations:** ^1^ Department of Bioinformatics and Genetics Swedish Museum of Natural History Stockholm Sweden; ^2^ Centre for Palaeogenetics Stockholm Sweden; ^3^ Department of Zoology Stockholm University Stockholm Sweden; ^4^ Biodiversity and Sustainability Solutions ‐ BaSS, Aveiras de baixo Lisbon Portugal; ^5^ Department of Medical Biochemistry and Microbiology, SciLifeLab Uppsala University Uppsala Sweden; ^6^ SciLifeLab Stockholm Sweden

**Keywords:** biodiversity monitoring, bulkDNA, k‐mer classification, metabarcoding, metagenomics

## Abstract

Obtaining genome‐wide data from complex samples, such as environmental material or bulk species collections, is increasingly feasible, yet inferring species presence and population genomic insights remains challenging. We applied metagenomic sequencing to 40 arthropod bulk samples collected with Malaise traps across Sweden and compared results with metabarcoding of the same material. Using a custom genome database, we achieved genus‐level classification largely consistent with metabarcoding. While metagenomics detected all genera identified by metabarcoding, conservative filtering thresholds designed to minimise false positives also excluded some true signals, particularly for low‐abundance taxa. Taxonomic overlap between methods was further constrained by limited reference database representation. Beyond taxonomic assignment, metagenomic sequencing yielded genome‐level information: we inferred haplotype diversity, heterozygosity and geographic population structure for several abundant species, including variable degrees of hybrid origin in red wood ants and the genetic distinctiveness of Gotland bumblebees. Finally, by‐catch plant DNA present in the bulk samples revealed plausible arthropod–plant interactions, several of which align with known ecological associations. Together, these results demonstrate the potential of metagenomics for biodiversity monitoring and population genomics, while underscoring the importance of filtering criteria and comprehensive reference databases.

## Graphical abstract

We used metagenomic sequencing of 40 bulk arthropod samples collected across Sweden to classify taxa and compare results with metabarcoding from the same samples. While metabarcoding was more sensitive for detecting low‐abundance taxa, taxonomic overlap between methods was strongly influenced by reference database representation. Metagenomics achieved genus‐level classifications broadly consistent with metabarcoding and further provided genome‐level insights into population structure, genetic diversity and hybridisation, as well as plausible arthropod–plant interactions through by‐catch DNA.

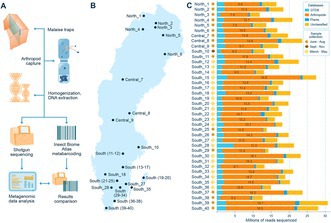

## 1 Introduction

**FIGURE 1:**
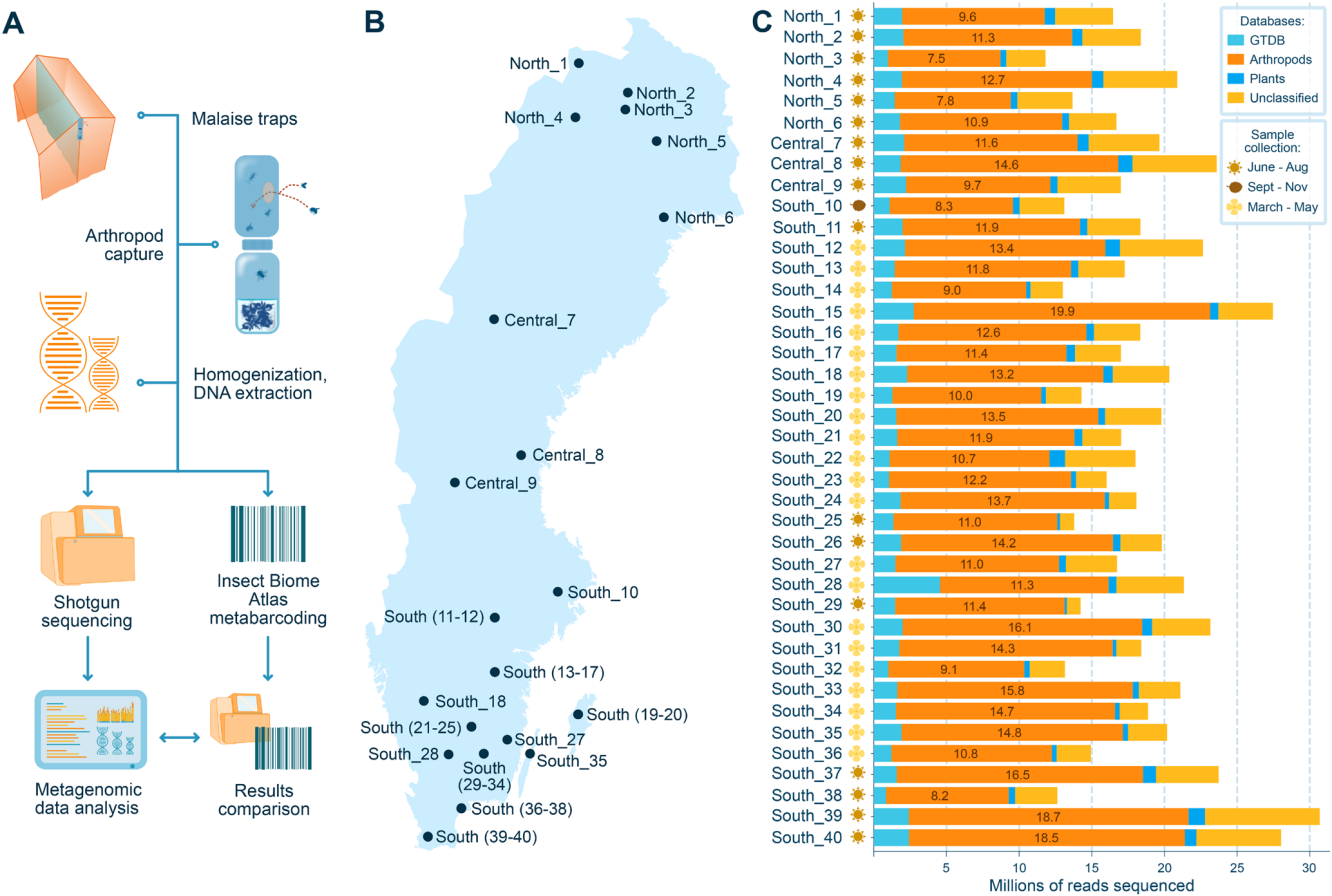
Overview of the conducted workflow, sampling locations and initial classification results. (A) Graphical representation of the steps followed prior to bioinformatic processing of the sequencing reads. From top to bottom it depicts the Malaise trap, the insect capture, the homogenisation and DNA extraction, the shotgun sequencing and the subsequent metagenomic and metabarcoding data analysis. (B) Map of Sweden with the names and locations of all 40 samples collected for this study, numbered from 1 to 40 and prefaced by ‘North’, ‘Central’ and ‘South’, depending on their geographic location. (C) Stacked bar plot showing millions of reads sequenced (*x*‐axis) for each sample (*y*‐axis) that were classified to the GTDB (bacterial and archaeal) database in light blue, the arthropod database in orange, the plant database in dark blue and unclassified reads in yellow.

The Malaise trap, invented by Swedish entomologist René Malaise (Malaise 1937), is one of the most widely used insect collection methods (Vårdal and Taeger 2011). The traps are tent‐like structures (Figure 1A) with mesh walls to which the organisms typically fly or walk against and move upwards into a single exit to a jar containing ethanol. Although usually associated with the word ‘insects’ (i.e., organisms that belong to class Insecta), Malaise traps can also capture other arthropods (i.e., from phylum Arthropoda) like spiders, mites and crustaceans (Malaise 1937). Many individual arthropods can be collected in a single trap over the period of a few days or weeks. Originally designed to expand museum collections, they are also ideal for the recovery of arthropod DNA.

The DNA extracted from mixed samples of different organisms, such as Malaise‐trap catches, is referred to as bulkDNA (Couton et al. 2023; Jerney et al. 2023). Determination of species composition using bulkDNA, as with environmental DNA (eDNA), presents technical and bioinformatic challenges due to its inherent complexity; the DNA stems from various biological sources within a single sample. In contrast to eDNA obtained from biological material shed by organisms into the environment, when extracting bulkDNA, the entire specimen is used (Couton et al. 2023). Thus, bulkDNA likely contains more DNA sequences that have not been exposed to degrading elements outside of the cell, potentially increasing its robustness for genetic studies.

Two approaches commonly used to determine species composition in mixed DNA samples are metabarcoding and metagenomics. The former relies on the Polymerase Chain Reaction (PCR) to amplify many copies of one or more known, short sequences of highly informative DNA for species identification, referred to as barcodes (Hebert et al. 2003). One of the most used barcodes across many life‐forms, including arthropods, is the mitochondrial protein complex cytochrome c oxidase 1 (CO1) (Kocher et al. 1989). Through the algorithmic clustering of existing barcodes obtained from thousands of specimens, experts establish Barcode Index Numbers (BINs) that are generally consistent with species classifications. Currently, 800,000 arthropod CO1 BINs are available in the Barcode of Life Data System, BOLD (Ratnasingham and Hebert 2013).

Metabarcoding is cost‐effective as it allows for the processing of large numbers of samples and the detection of up to hundreds of thousands of insect species (Yu et al. 2012). While metabarcoding methods revolutionised our ability to identify species from bulkDNA samples, one of its limitations is amplification bias: the preferential amplification of some DNA fragments present in the sample over others due to sequence composition, length, or abundance differences (Krehenwinkel et al. 2017), which can lead to species dropout and skewed abundance estimates (Nichols et al. 2018; Jerney et al. 2023). Another limitation of metabarcoding, common to all methods relying on large reference databases, is the varying levels of database curation (McGee et al. 2019; Ruppert et al. 2019; Weigand et al. 2019; Keck et al. 2023). Although metabarcoding data from bulkDNA has successfully been used to identify different haplotypes (Shokralla et al. 2014; Callahan et al. 2017; Elbrecht et al. 2018; Antich et al. 2022), using a single DNA marker limits the ability to infer within‐species diversity, including measures of population structure or geographic origin (Elbrecht et al. 2018).

Metagenomics, by contrast, is not based on a single region of the DNA; instead, a random subset of the entire DNA content is sequenced. Subsequent matching of the sequences against a reference genome database allows for the detection of species presence and the identification of genetic variants across the obtained genomes. Thus, contrary to metabarcoding, metagenomics can in theory provide insights into genetic diversity, population structure, admixture, gene‐flow patterns and selection processes, important metrics for conservation and evolution research (Parsons et al. 2018; Dugal et al. 2022; Farrell et al. 2022).

As powerful as this approach is, metagenomics is not without its limitations. The availability of large computing infrastructure is required as individual reference genomes can be billions of base pairs in size (Sessions 2013), resulting in computationally intensive sequence matching and alignments. Multiple tools have been developed with the aim to overcome these computational hurdles (Wood and Salzberg 2014; Lu et al. 2017; Breitwieser et al. 2018; Wood et al. 2019; Pockrandt et al. 2022; Pochon et al. 2023). A widely used tool, Kraken2 (Wood et al. 2019), for example, instead of finding matches across long regions of DNA, uses short nucleotide fragments usually around 31 bp long called minimisers. All unique minimisers in a set of reference genomes are stored in a hash table which is used to search for matches between the references and the sequence data. The software is phylogeny aware, with the minimisers classified to specific nodes in the tree. If, for instance, a minimiser matches to the genomes of multiple species within a genus, it is reported at the genus level (Wood et al. 2019).

The second limitation in a metagenomic approach is the availability of reference genomes. If a species is present in the analysed DNA data, but its reference genome is not available, species identification is not possible. Conversely, if a genetically closely related sister species is present in the reference database, the results might show a false positive signal, as the high proportion of shared DNA between the species will result in erroneously classified minimisers. Like with metabarcoding, careful curation of the used database is paramount to avoid classification errors (McGee et al. 2019; Ruppert et al. 2019; Weigand et al. 2019; Keck et al. 2023).

In this study, we subjected 40 bulkDNA samples from the Insect Biome Atlas projects (Miraldo et al. 2025) obtained from Malaise traps throughout Sweden to metabarcoding and metagenomics sequencing for taxonomic composition and population genetic analysis (Figure 1). The samples were particularly well suited for comparing methodological approaches for two key reasons. First, bulkDNA, through the extraction of genetic material from the specimen directly in the sample, offers both biological complexity and high DNA yields. Second, although arthropods have relatively large genomes (ranging from 90 to 7500 Mb (Hanrahan and Johnston 2011)), their genome sizes are still manageable for the creation of a comprehensive database containing complete arthropod reference genomes. We constructed a terabyte‐scale Kraken2 database and inferred species composition across the 40 bulk samples through large‐scale k‐mer classification. We compared these inferences with species detections obtained via metabarcoding on the same sample set (Miraldo et al. 2025). We also made our arthropod Kraken2 database publicly available for the wider community to use.

## 2 Materials and Methods

### 2.1 Sample Collection and Processing

The 40 Malaise trap bulk samples in this study (Figure 1B) were selected among the ~4800 samples collected by IBA (Miraldo et al. 2025) according to community composition similarities. First, all 7000 IBA samples were clustered based on the arthropod abundance estimates from the metabarcoding data (Miraldo et al. 2025) using non‐metric multidimensional scaling (using 5 dimensions and 1000 iterations). We then projected the samples across the first two dimensions and constructed a 2D density kernel across these points. The probability of a sample being selected was then proportional to its position across the 2D density, i.e., samples closer to the centre of the cluster density (and therefore more similar in composition) were more likely to be selected.

Contents of the Malaise trap samples were preserved in 95% ethanol before processing in the laboratory. The locations and sampling dates of the samples are described in Table S1. We manually added individuals from six insect species (from both commercial and laboratory strains, see Table S2) not naturally found in Sweden (*Drosophila serrata n* = 1, *D. jambulina n* = 1, *D. bicornuta* *n* = 3, *Shelfordella lateralis n* = 2, *Gryllodes sigilatus n* = 2 and *Gryllus bimaculatus n* = 1) as biological spike‐in controls (Table S2).

All 40 samples were subjected to two procedures before DNA extraction: a non‐destructive lysis (Iwaszkiewicz‐Eggebrecht, Łukasik, et al. 2023), and a mechanical and chemical homogenisation (Persson et al. 2024; Miraldo et al. 2025). This study focused on the homogenate results, although lysate comparisons are described in Appendix I. For the homogenisation procedure, the preservative ethanol was decanted, and each sample was ground up using the Ultra Turrax Drive homogeniser (IKA). The homogenate was additionally digested with a lysis buffer and proteinase K for 2 h 45 min at 56°C in a dry shaking incubator. We then added a standardised amount of two synthetic oligonucleotide sequences (synthetic spike‐ins) to each homogenate aliquot (Iwaszkiewicz‐Eggebrecht, Prus‐Frankowska, and Łukasik 2023), which served as positive sequencing controls but were not further used for downstream analysis in this study. During this stage of the large‐scale processing of the hundreds of IBA samples, 152 negative controls (19 with and 133 without the synthetic spike‐ins) were included (Miraldo et al. 2025).

DNA was purified from 225 μL of homogenate using silica‐coated magnetic beads with the KingFisher Cell and Tissue DNA kit on a KingFisher Flex 96 robot (both Thermo Scientific) according to manufacturer instructions (Iwaszkiewicz‐Eggebrecht, Łukasik, et al. 2023). Another 65 negative controls were used during the DNA extraction stages of the large‐scale sample processing (Miraldo et al. 2025).

For metabarcoding, amplicon libraries were generated targeting a 418‐bp fragment of the standard barcoding region of the cytochrome *c* oxidase subunit 1 (COI) gene using broad‐spectrum primers BF3 CCHGAYATRGCHTTYCCHCG (Elbrecht et al. 2019) and BR2 CDGGRTGNCCRAARAAYCA (Elbrecht and Leese 2017), together with 5′‐end Illumina sequence adapters (forward: ACACTCTTTCCCTACACGACGCTCTTCCGATCT‐3′, reverse: 5′‐GTGACTGGAGTTCAGACGTGTGCTCTTCCGATCT). Large‐scale sample processing included an additional 64 negative PCR controls. The PCR conditions used were: 95°C for 15 min, 25 cycles of 94°C for 30 s, 50°C for 90 s and 72°C for 90 s, with a final elongation step of 72°C for 10 min. PCR products were cleaned using magnetic beads (Carboxyl‐modified Sera‐Mag Magnetic Speed‐Beads, Hydrophobic, CYTIVA), with a 2:1 bead to sample ratio. An indexing PCR was conducted such that no reverse or forward index was used more than once on a single sequencing lane. Conditions for the indexing PCR were: 95°C for 15 min, 7 cycles of 94°C for 30 s, 50°C for 90 s and 72°C for 90 s, followed by a final elongation step of 72°C for 10 min. Samples were pooled and cleaned using the Promega ProNex Size‐Selective Purification System, with 1/1.5 (v/v) pool to magnetic beads ratio. Sequencing was carried out on an Illumina NovaSeq 6000 platform using SPrime 500‐cycle flow cells (Iwaszkiewicz‐Eggebrecht, Łukasik, et al. 2023).

For metagenomic analysis, shotgun sequencing libraries of the samples (homogenates and lysates) were prepared from the extracted DNA using the Illumina TruSeq DNA PCR‐Free Library Preparation Kit, following the manufacturer's standard protocol (Illumina, San Diego, CA, USA). Library preparation used 100 ng of DNA per sample, quantified using a Qubit dsDNA HS Assay Kit (Thermo Fisher Scientific) and assessed for integrity using a TapeStation 4200 system (Agilent Technologies). DNA was first fragmented to an average insert size of ~350 bp using a Covaris sonicator (Covaris Inc.) under the recommended conditions for PCR‐free library preparation. The fragmented DNA was then subjected to a series of enzymatic steps, including end‐repair to generate blunt ends and A‐tailing to add a single adenine overhang at the 3′ ends, facilitating adapter ligation. Illumina TruSeq single‐index adapters were ligated to the DNA fragments using a DNA ligase enzyme. After ligation, the adapter‐ligated DNA was purified using AMPure XP beads (Beckman Coulter) to remove unligated adapters, primer dimers and small DNA fragments. No PCR amplification step was performed, in accordance with the PCR‐free protocol, to minimise amplification bias and preserve the original fragment composition of the metagenomic DNA.

Library concentrations were evaluated using the Qubit. Fluorometric and equimolar amounts of each library were pooled and submitted for sequencing. Sequencing was performed on a single Illumina NovaSeq 6000 S4 flow cell, generating 150 bp paired‐end reads. Base calling and de‐multiplexing were conducted using Illumina's bcl2fastq software (v2.20), producing demultiplexed FASTQ files for downstream analysis.

As the 40 samples in this study were processed as part of the larger Insect Biome Atlas project, which included hundreds of samples processed in parallel, our focal 40 samples were not processed with dedicated negative controls. However, in total, the Insect Biome Atlas did produce 281 negative controls throughout the DNA processing procedures.

### 2.2 Taxonomic Classification of Metabarcoding Data

Sequencing reads of metabarcoding data were processed within the large‐scale analysis of IBA samples (Miraldo et al. 2025) using a Snakemake workflow (Sundh et al. 2021) that uses Cutadapt (v3.1) (Martin 2011) in four main steps: (1) removal of reads with Illumina TruSeq adapters at either end, (2) trimming of primer sequences from the start of R1 and R2 using exact matches (‐‐no‐indels ‐e 0), with untrimmed reads discarded, (3) removal of any remaining primer‐containing reads and (4) trimming reads to a fixed length based on the expected read length, minus the longest primer (default: 251 nt). Filtered reads were retained if 403 to 418 nt long (in 3‐nt steps) and lacked in‐frame stop codons.

Denoising was performed using the nf‐core/ampliseq Nextflow pipeline (v2.4.0) (Straub et al. 2020), which applies DADA2 to infer amplicon sequence variants (ASVs). ASVs were then processed with the HAPP pipeline (Sundh et al. 2024) for taxonomic annotation (via SINTAX (Edgar 2016) in VSEARCH (Rognes et al. 2016)), chimera removal, OTU clustering and filtering of NUMTs and noise. Taxonomy was assigned against a custom built (December 2022) COI reference database (Sundh 2023) from all BOLD sequences labelled as ‘COI‐5P’ and associated with BINs. Sequences were cleaned of gaps and ambiguous characters and clustered at 100% identity per BIN with the coidb package (Sundh 2024). As some BINs lack assignments at certain taxonomic levels, the ranks were filled in using the last known higher rank, adding an _X, _XX, etc., to indicate how far down the hierarchy the data is missing (Sundh 2024) (e.g., ‘Hominidae_X’ in the genus column and ‘Hominidae_XX’ in the species column would indicate the BIN was assigned to the family level Hominidae, but lacked a further genus and species assignments). This placeholder allowed the structure in the database to be preserved despite incomplete taxonomy.

ASVs classified as Insecta or Collembola but lacking order‐level assignment were placed phylogenetically with EPA‐NG (Barbera et al. 2019) and annotated with gappa (Czech et al. 2020), updating only the order‐level taxonomy. Chimeras were further removed with uchime (Edgar et al. 2011), and remaining ASVs were clustered using Swarm (v3.1.0, −d 15) (Mahé et al. 2014). Cluster taxonomy was assigned by weighted abundance consensus, i.e., names reaching ≥ 80% consensus at each rank were retained, and the rest were marked as ‘unresolved’ (Miraldo et al. 2025). Representative ASVs per cluster were selected based on the highest median read count across samples. Final noise and NUMT removal was done with NEEAT, considering taxonomy, co‐occurrence patterns, evolutionary signals and cluster abundance (Sundh et al. 2024). Clusters which lacked order‐level assignment, had less than three reads across datasets, or were present in more than 5% of the 281 sequenced blanks, were excluded (Miraldo et al. 2025).

In this study, we further converted the genus level data to presence (1.0) or non‐detection (0.0) in each sample using a threshold of 100 barcode reads. Genera that were annotated with ‘unclassified’, ‘_X’ or ‘_XX’ placeholders due to lack of taxonomic resolution for that BIN in the metabarcoding database were also removed due to insufficient taxonomic resolution.

### 2.3 Taxonomic Classification of Metagenomic Data

To identify the species origin of the metagenomic sequence data, we used the kmer classification tool Kraken2 (v2.1.2) (Wood et al. 2019). Three custom Kraken2 databases were constructed (kraken‐build, default parameters; see https://github.com/SamanthaLop/Small_Bugs_Big_Data for code used to prepare fasta files for database building). The first was a bacterial and archaeal database, built by Nikolay Oskolkov, with over 400,000 assemblies obtained from the Genome Taxonomy Database (GTDB, release 214), with each assembly representing a different species/strain (Parks et al. 2018, 2020, 2022; Rinke et al. 2021). Classification results from the GTDB database were used to screen samples for bacterial genomes frequently identified as contaminants in metagenomic and metabarcoding studies (Salter et al. 2014).

The second database built included all 2593 arthropod reference assemblies available on The National Center for Biotechnology Information (NCBI) as of March 2023 (Table S3) (Kitts et al. 2016). Of the four genera used as biological spike‐in controls, *Drosophila*, *Shelfordella*, *Gryllus and Gryllodes*, only the latter lacked a reference assembly and was thus not included in the arthropod database.

To explore the possibility of identifying plant DNA brought into the trap on the surface or in the gut of the arthropods, the third database in this study focused on plants. This database was built by (Jin et al., in preparation) and includes 1257 Viridiplantae NCBI genome assemblies (Kitts et al. 2016), all available RefSeq mitochondrial genomes (retrieved April 2023) (O'Leary et al. 2016) and assemblies for all 1323 species available in the PhyloNorway database (Alsos et al. 2020), which includes the majority of vascular plant species known to be present in the nordics. The plant database contains 2376 species from 960 genera in total (Table S4).

All fastq reads were trimmed of adapters and overlapping reads were merged using fastp (Chen et al. 2018). To increase accuracy, only the merged sequencing reads were then sequentially classified against the databases in the following order: GTDB (bacteria and archaea), arthropod and finally plants, using Kraken2 on default parameters and setting the flag—report‐minimiser‐data. Parsing and concatenation of Kraken2 classification output reports was done using a custom python module (kraken2_report_parsing_functions.py) available at https://github.com/SamanthaLop/Small_Bugs_Big_Data. Only the reads that could not be classified to the respective database served as the input data for the classification of the following database. This was done to minimise the number of false positive hits (e.g., bacterial reads that by chance also match conserved regions in the arthropod and plant genomes). Reads unable to be classified against any of the databases were deemed unclassified.

In an effort to reduce false‐positive arthropod classification, conservative filtering cutoffs were determined for each individual bulk sample by using the biological spike‐in genera present in the metagenomic database. In each sample, the lowest number of unique minimisers assigned to any of the three spike genera was determined, and all taxa with less than this sample‐specific minimal cutoff were deemed at too low an abundance to ensure reliable inference on their presence. Thus, a taxon was only considered as being present in the sample if it had higher or equal abundance of unique minimisers as the biological spike‐ins. To control for contamination, and given the lack of specialised blanks, we used the negative control data generated through the large‐scale IBA processing and made sure not to include genera with ASVs present in more than 5% of the negative controls (i.e., detected in 14 blanks or more).

### 2.4 Comparison of Metabarcoding and Metagenomic Sequence Data

Using the sample specific thresholds, the metagenomic data was binarised into presence (1.0) and non‐detection (0.0) records for each genus in the arthropod database. The metabarcoding data was binarised using a 100 read minimum for each species to confirm presence (1.0) or non‐detection (0.0). Comparisons of presence/non‐detection between the two datasets were then restricted to genera that were included in both the metagenomic and metabarcoding reference databases and plotted as a heatmap. We calculated, for every genus per sample, how often both genera overlapped between methods. This calculation was done using data filtered with the spike‐in sample‐specific filtering, as well as two other thresholds (500,000 and 100,000 unique minimisers).

### 2.5 Population Genomics of Metagenomic Data

Output generated by Kraken2 contains, for each node in the database (e.g., family, genus, species), the total number of unique 31 base pair matches (minimisers) between the sequence data and the reference assemblies constituting that node. While not an exhaustive search, we conducted a practical selection of taxa to demonstrate the possibilities of bulkDNA for population genomics; taxa with a high representation of unique minimisers in our Kraken2 results and good availability of public genomic resequencing data were prioritised. We first assessed genera with the highest number of both assigned unique minimisers and sequencing reads across all bulkDNA samples and then selected species within these genera with at least 20 or more publicly available shotgun genomes, ideally from Europe or Northern Europe. This selection led us to focus on three taxa: *Bombus pascuorum* (the common carder bee), *B. terrestris* (the buff‐tailed bumblebee) and the red wood ant hybrid *Formica aquilonia × F. polyctena*.

We aimed to obtain population genomic insights for these three taxa using the bulkDNA sequence data, as well as previously published high‐coverage sequenced genomes. We refer to the data obtained from mapping a bulkDNA sample to a reference assembly as a genome, even though the exact number of specimens being mapped is unknown (given the homogenisation of Malaise trap contents). First, we built a concatenated *Bombus* reference with all 33 *Bombus* reference assemblies (accessions available in Table S5) available on NCBI, which was then indexed and used to map all reads (including unmerged reads) of each bulkDNA sample using Bowtie2 on the—very‐sensitive setting (Langmead and Salzberg 2012). This concatenated *Bombus* reference was used to ensure that only reads specifically aligning to the *B. pascuorum* and *B. terrestris* assemblies were kept, whereas reads from different *Bombus* species or those reads matching conserved regions of multiple *Bombus* genomes were filtered out. For *Formica* population genomic insights, all reads (including unmerged reads) were mapped to a concatenated *Formica* reference containing the *F. aquilonia* × *F. polyctena* hybrid assembly and all other *Formica* reference genomes available on NCBI (*F. excecta*, *F. selysi* and *F. aserva* ; accessions in Table S6). Note that the *F. aserva* assembly became available only in March 2024, so while it was included in this mapping step, it was not part of the metagenomics database, built a year earlier. Coverages for the *Bombus* and *Formica* genomes were calculated using samtools (v1.20) (Li et al. 2009) depth.

Next, to investigate if we could detect the presence of multiple haplotypes within a single sample (as they would suggest multiple individuals of the same species being present), we selected two bulkDNA samples with the highest sequencing coverages for mitochondrial allele frequency analysis: *Bombus pratorum* from sample South_34 (20X coverage) and *Bombus pascuorum* from sample Central_8 (4.4X coverage). We used bcftools mpileup (Danecek et al. 2021) and a custom python script (mitochondrial_allele_frequencies.py, available at https://github.com/SamanthaLop/Small_Bugs_Big_Data) to extract per‐base allele frequencies across the mitochondrial genomes. As the number of individuals contributing to each sample is unknown, we compared the observed allele frequency distributions to distributions generated from datasets with a known number of individuals. As whole‐genome sequencing data for *B. pratorum* was not available, we used publicly available *B. pascuorum* genomes (Liu et al. 2024). One genome was selected from each of the nine distinct sampling localities, and these were combined into simulated pools representing increasing numbers of individuals (Table S7). All preprocessing, mapping and mitochondrial allele frequency calculations were conducted in the same way as the bulkDNA samples.

For population genomic insights, bulkDNA genomes of *B. pascuorum* , *B. terrestris* and *F. aquilonia × F. polyctena* with over 0.1X coverage were selected to obtain broader population structure insights (Lou et al. 2021). We also collected previously published high coverage genomes: 55 for *B. pascuorum* (Liu et al. 2017), 1738 for *B. terrestris* (Liu et al. 2017; Kawakami et al. 2019; Rahman et al. 2021; Barribeau et al. 2022; Colgan et al. 2022; Kardum Hjort et al. 2022; Larragy et al. 2023) and 40 for *Formica aquilonia* × *F. polyctena* (Nouhaud et al. 2022) (Table S8). These were mapped to the concatenated references in the same way as the bulkDNA samples, and only those with at least 0.1X (Lou et al. 2021) coverage were included in downstream analyses. From the bulkDNA and published genomes, we then randomly sampled an allele at each covered site using ANGSD haplocall and the following parameters: ‐doCounts 1 ‐dohaplocall 1 ‐minMinor 1 ‐remove_bads 1 ‐minMapQ 10 ‐uniqueOnly 1 (v0.940) (Korneliussen et al. 2014). The obtained allele calls were converted to BED format and used to conduct Principal Component Analyses (PCA) for each taxon using PCA‐emu (v1.0) (Meisner et al. 2021) with a minor allele frequency cutoff of 5%.

*B. pascuorum* heterozygosities of the previously published genomes, as well as of the Central_8 bulkDNA genome, were further inspected using 10 kb windows with samtools mpileup and counting the number of sites where more than one allele was found among the mapped reads.

### 2.6 Plant Classifications

To explore the possibilities of identifying plant‐pollinator interactions from the bulkDNA metagenomic data, we further analysed the Kraken2 output obtained from the plant classification step. First, we filtered each plant species occurrence based on a minimum threshold of 5000 unique minimisers. To focus on insect‐plant symbiotic candidate species, we then narrowed our analysis to the 18 most abundant plants that are known to be pollinated by arthropods. Then, we created a co‐occurrence matrix between these plants and the 25 most abundant arthropod genera known to have interactions with plants (ArtDatabanken 2023), which was then visualised through a heatmap. A co‐occurrence took place when the plant species and the arthropod genus were found, both after filtering, in the same sample. To increase comparability between the co‐occurrences, a bar plot was attached to the heatmap showing the number of samples in which each arthropod species was found, as these can inflate co‐occurrence values. Finally, we used the UK‐based Database of Pollinator Interactions (DoPI) to search for reported plant‐pollinator interactions between the plant species and arthropod genera in the co‐occurrence matrix (Balfour et al. 2022).

### 2.7 Data Visualisation

All plots in the main manuscript were generated using Python 3.12.3, and the following packages: Seaborn v0.13.2 (Waskom 2021), Matplotlib 3.9.2 (Hunter 2007), Pandas 2.2.3 (The Pandas Development Team 2025), Geopandas 1.0.1 (Jordahl et al. 2020), Shapely 2.0.6 (Gillies et al. 2025), Cartopy 0.24.1 (Elson et al. 2024) and SciKitLearn 1.7.1 (Pedregosa et al. 2012). Plots were exported as files in Scalable Vector Graphic (SVG) format from Python and imported in Affinity Designer 2.5.7 to generate the final figures. The shapefile used to represent the map of Sweden in Figure 1 was obtained from geoBoundaries (Runfola et al. 2020).

## 3 Results

### 3.1 Sample Collection and Processing

The DNA extracted from the 40 homogenised samples (Table S1) yielded concentrations between 1.53 and 36 ng/μL per sample. Metabarcoding sequencing resulted in an average of 29,000 barcode reads per sample, ranging from 5807 to 642,255, while metagenomic sequencing resulted in an average of 68 million reads per sample, ranging from 43 to 111 million reads (Figure 1C, Table S9).

### 3.2 Taxonomic Classification of bulkDNA

In total, we detected 5209 ASV clusters in the metabarcoding sequence data, corresponding to 1627 different genera (excluding the spike‐in genera) identified using our custom BOLD database (Table S10). The 100 barcode read threshold used for filtering reduced this number to 1191 genera and allowed for the conversion of values to 1.0 (presence) and 0.0 (non‐detection). However, 81 of these taxa corresponded to those BINs with insufficient taxonomic information at the genus level or lower (labelled in the database with the highest known taxonomic rank and a placeholder suffix ‘_X’, ‘_XX’, etc.). Removal of these 81 taxa resulted in 1110 genera, of which only 171 were present in both the metagenomics and metabarcoding databases (Table S11), and thus were used in further comparisons between both approaches.

Blanks used in the large‐scale sample processing of the IBA (Miraldo et al. 2025) were reported to only have two ASVs occurring in over 5% of the total 281 blanks (Miraldo et al. 2025) (Table S12). The first was an ASV corresponding to *Homo neanderthalis*, the neanderthal, detected in 108 controls (or 38% of the blanks), signalling human contamination, as human data is not included in the BOLD database (Miraldo et al. 2025). The second was an ASV cluster matching *Salticus scenicus* , a zebra spider, found in 90 controls (equivalent to 32% of the blanks), which is a common species in the facility where the IBA samples were processed (Miraldo et al. 2025). The ASVs with the next highest prevalence across blanks were found in no more than 10 blank samples each (equivalent to approximately 1%–3% of blanks) and corresponded to biological spike‐in control species (*Drosophila serrata, D. bicornuta*, *Shelfordella lateralis* and *Gryllus bimaculatus*) and two fly species: *Hydrotaea* sp. and *Thricops cuncutans* (Table S12).

In the metabarcoding data, the genus *Salticus* was detected in only one of the 40 samples in this study (South 36), while *Hydrotaea* and *Thricops* were each detected in seven samples (Table S10). However, none of these genera were included in our arthropod reference database, as no published genome assemblies were available at the time. As a result, they did not affect the interpretation of our metagenomic results.

On average, 9.46% of the metagenomic reads were classified to the GTDB database, with proportions ranging from 6.08% to 21.4% across samples (Figure 1C, Table S9). Further inspection revealed that an average of 1089 reads (< 0.001% of all sequenced reads) were classified to known bacterial contaminants (Salter et al. 2014). Only nine samples contained contaminant genera that had over 100,000 unique minimisers assigned (*Stenotrophomonas*, *Psychrobacter*, *Janthinobacterium*, *Enterobacter*, *Sphingobium* and *Escherichia*; Table S13; Appendix II), yet even across those samples and genera, the average number of reads classified was 2008 (< 0.002% of all sequenced reads). As these results did not indicate substantial contamination, no samples were removed from downstream analysis.

A total of 68.77% of reads could be categorised as arthropod‐derived, ranging from 54.37% to 81.94% between samples (Table S9). Of these, an average of 76.92% was assigned to species level. An average of 2.93% of the total merged reads could be classified to plant taxa, ranging from 1.11% to 5.94% between samples (Figure 1C, Table S9).

**FIGURE 2:**
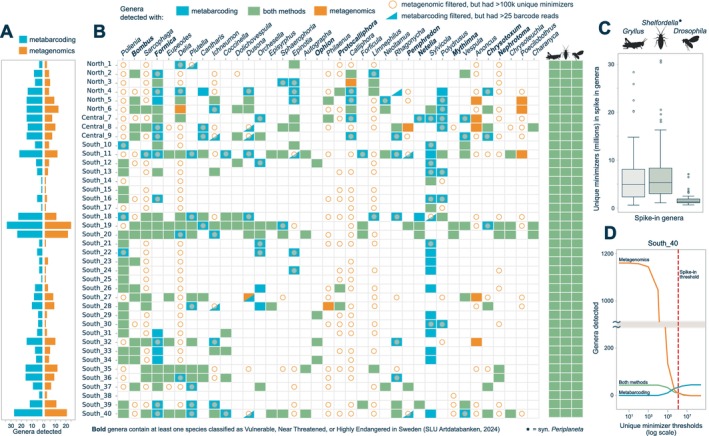
Taxonomic classification results with both methods. (A) Comparative barplot showing, for the 1191 genera present in both reference databases, the total genera detected with metabarcoding and metagenomics. (B) Heatmap showing post‐filtering presence/non‐detection data for the 40 genera (*x*‐axis) in each sample (*y*‐axis) that had the highest number of unique minimisers assigned. Additionally, orange‐coloured circles indicate instances with over 100 k classified unique minimisers but did not pass our sample‐specific spike‐in filtering step (any hits with less minimisers than the lowest spike‐in signal in the sample were removed), and blue triangles indicate it did not pass metabarcoding filtering (100 barcode reads minimum) but had more than 25 barcode reads present. The three most‐right columns show the three spike‐in genera present in the metagenomics database: Gryllus, Shelfordella (syn. Periplaneta) and Drosophila. Genus names in bold have at least one species classified as Vulnerable, Near Threatened, or Highly Endangered in Sweden (ArtDatabanken 2023). (C) Boxplot of the number of unique minimisers reported across all 40 samples for the three spike‐in genera. (D) Line graph of the number of genera detected (*y*‐axis) through metagenomics only, metabarcoding only, or through both approaches in sample South_40, with varying metagenomics filtering thresholds (*x*‐axis, log scale), the threshold that was implemented for the sample is represented by a dotted red line.

The metagenomics approach initially classified 1346 arthropod genera across all samples, although it contained a high number of false positive hits. After sample‐specific filtering and excluding the spike taxa (Figure 2C), 92 different genera remained, of which 90 are known to occur in Sweden (Vilkamaa 2000; ArtDatabanken 2023). The two detected genera not reported in Sweden are *Belgica* (a flightless midge genus) and *Schistocerca* (a grasshopper genus). The former was detected only in a single sample in northern Sweden (North_1) with 1.4 million unique minimisers. Meanwhile, *Schistocerca* was detected in two southern Sweden samples (South_39 and South_40), with 2.3 and 3.1 million unique minimisers, respectively. The total number of detected genera per sample ranged from three to 28, with the highest number in southern Sweden (Figure 2A).

### 3.3 Comparison of Metabarcoding and Metagenomic Data

At the genus level, the metabarcoding database included 48,009 known arthropod genera, while the metagenomic database included only 1357. The number of overlapping genera between both reference databases was 1216 (Table S14). In addition to the three biological control genera, both approaches detected 94 arthropod genera in all combined sequence data: 83 detected by both methods, and 11 with metagenomics only. Filtering thresholds had a substantial impact on the overlap between metabarcoding and metagenomics detections. Using spike‐in sample‐specific filtering, metagenomics detected a genus in 57.4% of the cases where metabarcoding did. Reducing the filtering threshold to 500,000 unique minimisers instead raised this overlap to 72.9%. When the threshold was further lowered to 100,000 and 10,000 unique minimisers, metagenomics detected a genus in a sample in 91.7% and 99.2% of the cases where metabarcoding did, respectively. Next, we explored overlaps and differences in the most abundant genera detected with both approaches (Figure 2B).

The presence of the 11 genera identified only through metagenomics was, after further investigation, found to be most likely due to a lack of appropriate representation in the metagenomic database. In these cases, the biological signal corresponded to genera not present in the reference database, and sequence reads were thus classified to those phylogenetically related genera that did have a reference assembly in the database.

Further analysis showed that, although not passing our sample‐specific filtering step, all genera and many of the genus‐sample occurrences identified exclusively through metabarcoding also had unique minimisers assigned to them in the metagenomic analysis (Figure 2B).

When differentiating between detected and non‐detected genera through the metagenomics approach, we found that non‐detected genera also contained significantly fewer metabarcoding reads (*t*‐test, *p* = 9.3e−06; Table S10). This indicates that species at low abundance in the samples are more likely to be missed or filtered out from the metagenomic data, while the more sensitive metabarcoding method can still detect the signal. As many of the genera not detected in the metagenomic data had unique minimisers assigned to them, our conservative threshold designed to minimise false positives likely also resulted in the exclusion of a proportion of truly present genera (Figure 2D).

### 3.4 Population Genomics of Metagenomic Data

Among our samples, we detected several species with over one million unique minimisers assigned. These included, among others: the common carder bee ( *Bombus pascuorum* ), the buff‐tailed bumblebee ( *B. terrestris* ); the early bumblebee ( *B. pratorum* ), and a hybrid red wood ant product of two species of the *Formica rufa* group ( *F. aquilonia* × *F. polyctena* ). Mapping of reads to the concatenated *Bombus* and *Formica* references resulted in coverages of up to 4.4X for the common carder bee ( *B. pascuorum* ); 3.2X for the buff‐tailed bee ( *B. terrestris* ); 1.8X for the European redwood ant species *F. polyctena* and *F. aquilonia* ; and even a 20X coverage genome for the early bumblebee ( *B. pratorum* ).

**FIGURE 3:**
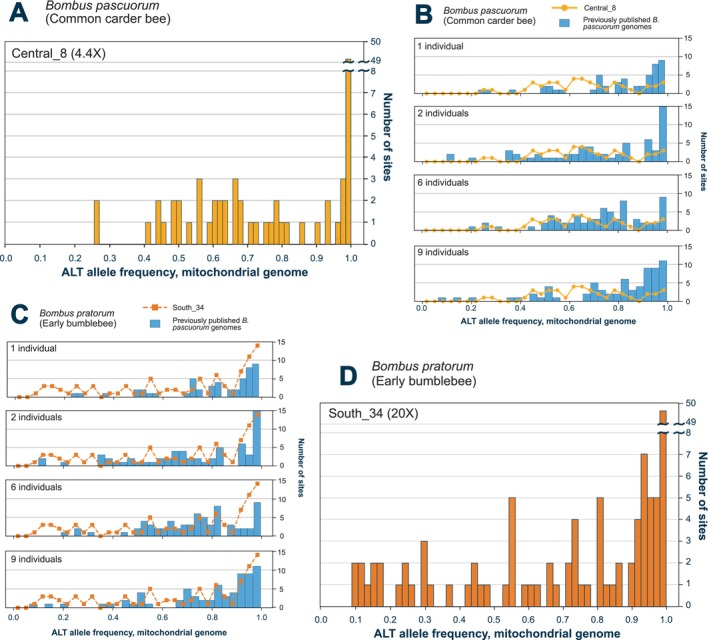
Mitochondrial genome allele frequency distributions in *Bombus pascuorum* and *Bombus pratorum* . (A) Histogram of alternative allele frequencies across the mitochondrial genome of *B. pascuorum* from the bulkDNA sample Central_8. (B) Four histograms showing alternative mitochondrial allele frequencies from previously published *B. pascuorum* genomes. Each plot displays blue bars representing allele frequencies < 1.0 for concatenated genome sets with 1, 2, 6 and 9 individuals. The yellow line, identical across all four plots, represents the allele frequency distribution of the *B. pascuorum* bulkDNA mitochondrial genome of sample Central_8. (C) Four histograms showing alternative mitochondrial allele frequencies from previously published *B. pascuorum* genomes. Each plot displays blue bars representing allele frequencies < 1.0 for concatenated genome sets with 1, 2, 6 and 9 individuals. The yellow line, identical across all four plots, represents the allele frequency distribution of the *B. pratorum* bulkDNA mitochondrial genome of sample South_34. (D) Histogram of alternative allele frequencies across the mitochondrial bulk genome of *B. pratorum* from the bulkDNA sample South_34.

The allele frequency distribution of the *B. pascuorum* mitochondrial genome from sample Central_8 (30.8X mitogenome coverage; Figure 3A) was relatively uniform, though the presence of multiple distinct peaks suggests more than one haplotype within the sample. In contrast, the *B. pratorum* mitochondrial allele frequencies from sample South_34 (183X mitogenome coverage; Figure 3D) displayed a more heterogeneous distribution, likely reflecting a greater number of haplotypes within the sample, and/or variable representation of each. Comparison with simulated distributions generated from pooled samples with known numbers of individuals (Figure 3B,C) revealed similar patterns to those expected for multiple individuals, although neither matched any simulated profile exactly. These results indicate the presence of several individuals with different haplotypes in both samples; however, accurately determining the exact number of individuals from allele frequency spectra remains highly challenging.

**FIGURE 4:**
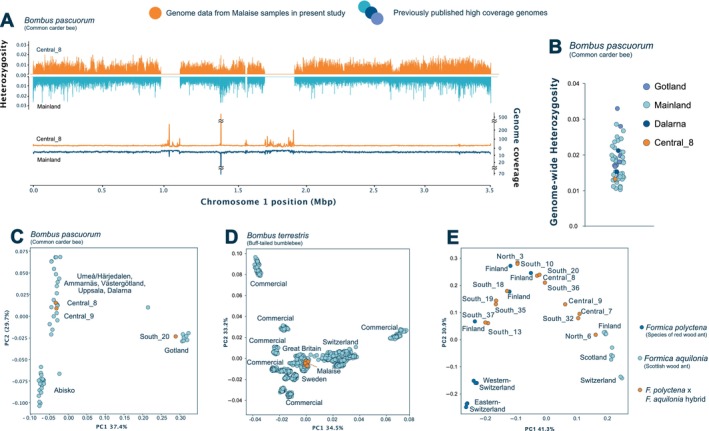
Population genomics analyses of selected taxa. (A) Mirrored heterozygosity bar plots comparing heterozygosities in Chromosome 1 from the *B. pascuorum* bulk genome from our metagenomic sample Central_8 (in orange) to a previously published genome from the mainland (in blue). Below, with the same colour code, are mirrored line plots showing the coverage in the same positions for both genomes. A coverage peak, likely due to the centromere, is observed between positions 1.0 and 1.5 Mbp in both genomes. (B) Dot plot showing the heterozygosity of the *B. pascuorum* Central_8 bulk genome in orange, with the heterozygosity of previously published genomes from across Sweden in different blue tones. (C) Principal Component Analysis plot showing three of the *B. pascuorum* bulk genomes obtained in the current study (orange) and other previously published genomes from across Sweden (blue). (D) PCA plot showing our obtained *B. terrestris* bulk genomes from 10 samples (orange), and a set of previously published genomes for the same species (blue). (E) PCA plot showing the obtained Formica bulk genomes (orange) along with previously published genomes (blue tones).

Further analysis gave us insights into its genome‐wide heterozygosity, which we compared to a previously sequenced, high‐quality genome from Sweden (Figure 4A,B). We find that heterozygosity and coverage across the genome is highly similar to a previously sequenced individual from the same region (Figure 4A) and its genetic diversity clusters among other previously sequenced *Bombus pascuorum* genomes (Figure 4B), supporting the accuracy of the measured statistics. Using a set of previously sequenced genomes, we obtained insights into the population structure by conducting a PCA on the identified genomic variants, placing our bulkDNA into geographic patterns (Figure 4C,D). We were able to confirm the geographic origin of the common carder and buff‐tailed bee genomic data in three and ten of our samples, respectively, which matched the genomic profile of previously collected genomes from the same areas, including confirmation of a unique genomic population in Gotland, an island on the Swedish east coast.

PCA analyses from the *F. polyctena* × *F. aquilonia* hybrid species complex, which naturally hybridises in Sweden, provided two main findings. First, while geographic resolution was somewhat limited due to the lack of other sequenced genomes from Sweden, our samples clustered more closely to published genomes from Finland than to those from Switzerland or Scotland (Figure 4E). Second, on top of the geographic clustering, the PCA revealed a hybrid continuum in our samples between the two species, with genomes likely distributed according to the fraction of ancestry of both species in their genome (Figure 4E).

### 3.5 Plant Classifications

**FIGURE 5:**
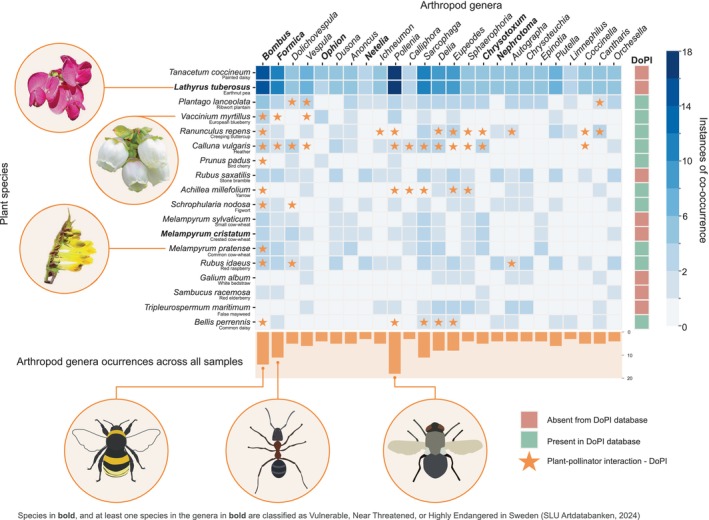
Co‐occurrence heatmap between classified plant species and selected arthropod genera known to have close interactions with plants. Co‐occurrences ranged from zero (no samples contained both taxa) to 18 (18 samples contained both taxa). The right‐most column named indicates if the plant species of that row is present in DoPI, the Database of Pollinator Interactions (Balfour et al. 2022). Stars in the heatmap indicate confirmed plant‐pollinator interactions between a plant species and arthropod genus reported in the DoPI. Below the heatmap is also a bar plot of the total occurrences of those insect genera across all samples [Images of earthnut pea and European blueberry adapted from photographs by Cornel Frühauf and Stephan (Pixabay, public domain); photograph of common cow wheat and arthropod illustrations by first author].

We also explored the possibility of detecting interactions between plants and insects from bulkDNA metagenomic data, by matching our data to a large custom‐built plant database, containing, among others, skimmed genomes from nearly all Scandinavian plant species (Kitts et al. 2016; O'Leary et al. 2016; Alsos et al. 2020). We focused on the plant species with the highest number of unique minimisers assigned. While many of the detected plant species are wind pollinated, i.e., *Pinus* spp. (pine), *Larix* spp. (larch), *Picea* spp. (spruce) and *Cupressus* spp. (cypress), we also identified several plant taxa known to occur in Sweden and to have a close ecological relationship with insects (Figure 5).

Of the 25 most abundant arthropod genera known to have interactions with plant species, three in particular were found to co‐occur (i.e., both were detected in the same sample) in the data together with plants known to interact with arthropods (Figure 5): *Bombus* (bumblebees), *Formica* (ants) and *Pollenia* (cluster flies). Three species of the hemiparasitic cow‐wheat genus *Melampyrum*, including the near threatened crested cow‐wheat (SLU Artdatabanken 2020), were detected and found to be co‐occurring in the sample with its known pollinators like *Sphaerophoria* hoverflies (Haug 2017) and the wasp‐mimicking *Chrysotoxum* (SLU Artdatabanken 2020). Other notable plant taxa found co‐occurring with arthropods in the same sample were, among others: blueberry ( *Vaccinium myrtillus* ), with bumblebees, wasps, flies and cluster flies; buttercup ( *Ranunculus repens* ), with bumblebees, ants, wasps, flies, cluster flies, crane flies, moths, caddisflies and soldier beetles; and red raspberry ( *R. idaeus* ), with bumblebees, ants, wasps, flies, cluster flies, hoverflies, craneflies, moths, ladybirds and springtails (Figure 5). We additionally used the Database of Pollinator Interactions (DoPI), which documents British pollinator–plant associations, to validate the identified co‐occurrences of plant and arthropod DNA (Balfour et al. 2022). For 10 of the 18 species, the DoPI database contained relevant records, and several of the co‐occurrences we detected have been previously reported as plant–pollinator interactions (Figure 5).

## 4 Discussion

The aims of this study were to compare metabarcoding and metagenomic approaches for species identification in mixed samples and explore the possibilities of inferring population genetic insights from metagenomic data. The 40 bulkDNA samples collected from Malaise insect traps across Sweden were particularly well‐suited for these goals as they form a complex DNA mixture, have high concentrations of DNA, and are taxonomically diverse.

Despite the relatively large genome sizes of insects (Hanrahan and Johnston 2011), we successfully built a genome reference database of around 1.1 TB in size, containing all 2593 arthropod reference assemblies available at the time. While it allowed for taxonomic classification of bulk arthropod samples, the database only covered about 2% of all known arthropod species and 8% of the genera reported in Sweden (Backlund 2024). With this in mind, we carried out most of our analyses at the genus level to increase taxonomic representation and analytical resolution. However, limited taxonomic representation in reference assemblies remains a major challenge in metagenomic studies (Couton et al. 2023). Additionally, contamination during the assembly of arthropod and plant genomes can result in the unintended incorporation of bacterial sequences into those assemblies, meaning microbial DNA present in the bulkDNA samples may be incorrectly classified as a target taxon (Gruber 2015; Lu and Salzberg 2018; Oskolkov et al. 2025). This issue is further exacerbated by random sequence similarity between microbial genomes and those in our reference database, as well as by possible instances of horizontal gene transfer. To reduce misclassification caused by arthropod‐associated microbiome DNA, we first classified all merged sequencing reads against a custom‐built, comprehensive bacterial and archaeal reference database (Parks et al. 2022). This filtering step removed a substantial portion of microbial reads and significantly improved the accuracy of our downstream taxonomic and genomic inferences.

Our comparison of metabarcoding and metagenomics revealed four insights. First, taxonomic signals were broadly consistent across methods but highly dependent on reference database representation. Metabarcoding generally recovered more genera per sample than metagenomics, although relaxing the stringency of the metagenomics filtering increased the overlap between methods considerably. With custom spike‐in filtering, metagenomics detected a genus in 57.4% of cases where metabarcoding did; this increased to 91.7% with a 100,000 unique minimiser threshold, and to 99.2% with a 10,000 unique minimiser threshold. Careful consideration of filtering criteria is essential when comparing or integrating results from these two approaches: lowering filtering thresholds increases overlap considerably, although at the cost of increased false positives.

Second, our results highlight and support the well‐known importance of proper taxonomic coverage and reliable taxonomic annotation of the reference databases, which can affect both metabarcoding and metagenomics approaches (McGee et al. 2019; Ruppert et al. 2019; Weigand et al. 2019; Keck et al. 2023). In our study, the presence of some genera (*Belgica* and *Schistocerca*) in the metagenomic results despite their absence from known Swedish fauna was explained by their close proximity to common genera that lacked genome references. The sample in which *Belgica* was detected also contained large numbers of barcode reads for *Lymnophyes* (a genus in the same subfamily, Orthocladiinae) and other Chironomidae genera. Further, the only two samples where we detected *Schistocerca* were also the samples with large numbers of barcode reads for *Chorhippus* and, to a smaller extent, for *Omocestus*. Both *Chorhippus* and *Omocestus* belong to the same subfamily as *Schistocerca* (Gomphocerinae) but neither was represented with a reference assembly in our metagenomic database. Conversely, abundant taxa in metabarcoding, like *Sylvicola* (a type of wood gnats), were under‐detected in metagenomics, potentially due to reads being classified at higher taxonomic levels when no closely related genome was available, limiting the number of diagnostic minimisers. Similar mismatches were evident in other taxa, particularly in diverse and poorly sequenced groups like *Ichneumonidae* (Darwin wasps) (Quicke 2014).

Third, despite database limitations, a substantial proportion of the total diversity in the bulk samples was captured with metagenomics, albeit mostly at higher taxonomic levels (Figure 1C). On average, 69% of all metagenomic reads were classified against our arthropod database, with an additional 9.46% and 2.93% of reads assigned to microbial and plant taxa, respectively, and only around 18% of reads remained unclassified (Figure 1).

Fourth, metabarcoding displayed greater sensitivity for detecting low‐abundance taxa and taxon‐sample occurrences. This can be attributed to the sensitive PCR‐based methodology, since just a few target molecules are needed to enable detection. However, the conservative filtering criteria we implemented on the metagenomics data also played a role. When, for instance, assessing the number of genera that would be identified for a single sample (South_40) and varying degrees of stringency in the unique minimiser threshold, it becomes clear that while our chosen spike‐in filters solidify our certainty in the results and eliminate false positives, they also exclude true positive signals that were picked up by metabarcoding (Figure 2C).

While both approaches contributed valuable biodiversity insights, use of metagenomics allowed us to retrieve genome‐level data from the bulk samples. Sufficient coverage for some taxa allowed the analysis of mitochondrial allele frequency variation (Figure 3), heterozygosity and genomic diversity metrics (Figure 4). For example, genome‐wide heterozygosity estimates from a *B. pascuorum* bulkDNA genome matched those from a single sequenced individual from Sweden and fell within that observed for the whole species (Figure 4A,B).

A key consideration when interpreting genomic data from mixed samples like bulkDNA or eDNA is the potential contribution of multiple individuals per taxon. Since all arthropods in each Malaise trap in this study were homogenised prior to DNA extractions, parameters like heterozygosity, haplotype diversity and allele frequencies do not always reflect a signal from a single individual but can be a composite signal of multiple genomes and their relative contributions. While this does not invalidate the biological signal, it can influence the interpretation of genomic variation (Schlötterer et al. 2014; Sigsgaard et al. 2020). For example, elevated heterozygosity could result from pooling individuals with distinct haplotypes rather than reflecting within‐individual variation. While this limits precise individual‐level resolution and relies on the availability of well‐curated reference assemblies, it also presents an opportunity: obtaining a population‐level snapshot from complex samples without requiring individual sampling and/or extractions, which can be key for ecological monitoring and rare or cryptic taxa.

Estimates of species abundance from mixed samples are similarly complicated by the variability in biomass and body size. As our samples were not processed to control for differences in biomass among specimens, read counts alone, either from metagenomics or metabarcoding, do not provide a reliable estimate of individual abundance but rather broadly reflect biomass (Elbrecht and Leese 2015; Elbrecht et al. 2017; Lamb et al. 2019). We recommend that the use of metagenomic data for ecological and population genetic inference be further explored, while also considering several methodological considerations. For instance, homogenising all specimens within a sample without controlling for specimen size could bias results toward larger‐bodied or more abundant species. However, incorporating an extra step of sample sorting based on size may increase accuracy, but it also adds logistical complexity, especially when dealing with hundreds of samples.

Despite these methodological challenges, our results show how metagenomics data can enable population‐scale insights. This included georeferencing of samples based on genetic variant patterns in relation to other publicly available genomes of the same species across Sweden and Europe. For example, the *B. pascuorum* bulkDNA genome from sample South_20, collected on the Swedish island of Gotland, clustered clearly with other Gotland samples (Figure 4C). *B. pascuorum* bees are known to be genetically isolated from the mainland and have even been recognised as a distinct subspecies: *B. pascuorum gotlandicus* (*Prerevisional Checklist and Synonymy of the Bees of Sweden (Hymenoptera: Apoidea): Preliminary Report* 2003; Liu et al. 2024). The remaining *B. pascuorum* bulkDNA genomes from this study, all collected on the mainland, clustered accordingly with other mainland genomes. A similar pattern can be observed in the clustering of bulkDNA genomes of *B. terrestris* with other Swedish genomes for the same species to the exclusion of other European samples (Figure 4D). Although individual reference genomes for the two parental species of the red wood ant hybrid ( *Formica polyctena* and *F. aquilonia* ) were not available, our analysis using the hybrid red wood ant genome was also in line with geographical expectations. Our samples clustered more closely to previously published genomes collected in Finland than to those collected in Switzerland. Further, a clear genetic gradient between the two species, likely attributed to the varying degrees of hybrid origin of the individuals, could be distinguished in the clustering, showing how metagenomics data can offer direct insights into population ancestries (Figure 4E).

Beyond arthropod presence/non‐detection and population‐level insights, metagenomic data allowed us to explore insect–plant interactions through the plant genomic content in the bulkDNA samples (Figure 5). While plant DNA has previously been recovered from Malaise traps (Betts 2010; Köthe et al. 2023; Newton et al. 2023; Thomas et al. 2024), this has, to date, only been analysed through the use of short genetic markers. Here, we identified a significant fraction of reads from plant genomes. For instance, we detected three species of cow‐wheat (*Melampyrum* spp.), one of which is classified as near threatened (SLU Artdatabanken 2020), together with DNA from their known hoverfly pollinators (*Sphaerophoria, Chrysotoxum*) (Haug 2017), including red‐listed species (SLU Artdatabanken 2020). While not an exhaustive comparison, some of these co‐occurrences were supported by records from the Database of Pollinator Interactions (DoPI), which, although focused on British species, contains over 300,000 interaction records and provides additional context for the interpretation of co‐occurrences (Balfour et al. 2022). Bumble bees (*Bombus* spp.) were also found to co‐occur with several plants for which they are well‐known key pollinators, such as blueberries ( *Vaccinium myrtillus* ) (Bartholomée et al. 2024), heather ( *Calluna Vulgaris* ) (Mahy et al. 1998) and raspberries ( *Rubus idaeus* ) (Ryan 2023), among others.

In addition to the arthropod and plant classifications, an average of 9.5% of the reads in the bulkDNA samples were classified to a wide variety of bacteria and archaea taxa (Figure 1C). While not further explored in this study, this data can be used to study arthropod microbiome and/or other bacterial‐arthropod interactions (Ladin et al. 2021). This potential, together with the results obtained in this study, helps illustrate how entire community interactions can be obtained from metagenomic data.

## 5 Conclusions

This study demonstrates the possibilities of applying specialised bioinformatic tools combined with large genome databases to process eukaryote metagenomic data from bulkDNA samples. This adds to a growing number of studies (Parsons et al. 2018; Ladin et al. 2021; Banerjee et al. 2022; Dugal et al. 2022; Farrell et al. 2022; Andres et al. 2023; Hassan et al. 2023; Thomas et al. 2024) that highlight the potential of bulkDNA and eDNA samples for biodiversity monitoring and conservation genomics.

Our comparison between metabarcoding and metagenomics approaches showed that while metabarcoding remains more sensitive for detecting low‐abundance taxa, taxonomic overlap between both methods is heavily influenced by reference database representation and classification filters. By using metagenomics, we were able to classify the majority of the obtained sequencing reads to a particular taxon, suggest several putative plant‐insect interactions at the genus level, detect near‐threatened species, determine genetic diversity and infer population origin of bulkDNA genomes.

While this study made use of material from bulkDNA samples, the approach can be adapted to other samples of mixed nature, such as environmental or sedimentary DNA. We expect that with continued advances in specialised bioinformatic tools, broadening reference databases and wider access to high‐throughput sequencing technologies, the field of metagenomics will continue to evolve.

## Author Contributions

**Samantha López Clinton:** conceptualization (lead), data curation (equal), formal analysis (lead), investigation (lead), methodology (lead), visualization (lead), writing – original draft (lead), writing – review and editing (equal). **Ela Iwaszkiewicz‐Eggebrecht:** data curation (equal), formal analysis (supporting), investigation (equal), methodology (supporting), writing – review and editing (equal). **Andreia Miraldo:** conceptualization (supporting), funding acquisition (supporting), methodology (supporting), resources (supporting), writing – review and editing (supporting). **Robert Goodsell:** data curation (supporting), investigation (supporting), methodology (supporting), writing – review and editing (supporting). **Matthew T. Webster:** methodology (supporting), resources (equal), writing – review and editing (supporting). **Fredrik Ronquist:** data curation (equal), formal analysis (supporting), funding acquisition (equal), investigation (supporting), methodology (equal), project administration (supporting), resources (equal), supervision (supporting), writing – original draft (supporting), writing – review and editing (equal). **Tom van der Valk:** conceptualization (equal), formal analysis (supporting), funding acquisition (lead), investigation (equal), methodology (equal), project administration (lead), resources (lead), supervision (lead), writing – original draft (supporting), writing – review and editing (equal).

## Conflicts of Interest

The authors declare no conflicts of interest.

## Supporting information


**Data S1:** ece372163‐sup‐0001‐DataS1.xlsx.

## Data Availability

All metagenomic sequence data is available in ENA under project accession PRJEB78579. Metabarcoding sequence data is available on ENA under project accession PRJEB61109. All Kraken2 classification reports are available at https://palaeogenetics.com/data‐and‐scripts/. The Kraken2 arthropod genome database is publicly available on the Scilifelab DataPortal at https://doi.org/10.17044/scilifelab.29666605. R code used for metabarcoding and metagenomic statistical testing for genera shared between databases is publicly available on GitHub at: https://github.com/insect‐biome‐atlas/paper‐metagenomics. Tables S1–S14, and an Index describing their contents, are provided in an accompanying Excel file (XLSX) available with the published article.

## Appendix I Lysate Metabarcoding Results

During the large‐scale IBA processing (Miraldo et al. 2025), all samples also underwent a non‐destructive lysis procedure using the FAVIS protocol (Iwaszkiewicz‐Eggebrecht, Łukasik, et al. 2023), which involved a 2‐h 45‐min incubation in lysis buffer. DNA was then purified from a 225 μL aliquot of the lysate. Comparisons of these lysate‐based metabarcoding results to the metagenomics data are summarised here but were excluded from the main manuscript; both lysate and homogenate metabarcoding data are available in Table S10.

Metabarcoding of lysates resulted in an initial 5623 unique ASV clusters corresponding to 1655 genera, excluding the spike‐in genera. Filtering for a minimum of 100 barcode reads reduced this number to 1241. Of these genera, 102 lacked enough taxonomic information and were labelled in the database with either an ‘unclassified’ prefix or an ‘_X’ suffix, and the last known higher taxonomic level. Removal of these 102 taxa resulted in 1139 genera, of which 177 were present in both the metagenomics and metabarcoding databases, and thus used in the comparison of both approaches. Only one arthropod genus, *Salticus*, was detected in over 5% of the IBA blanks (Miraldo et al. 2025); nevertheless, its removal from downstream analyses was not needed, as it had already been excluded due to being absent from the metagenomics assembly database.

One of the eleven genera detected exclusively through metagenomics, *Pemphredon* (digger wasps), was initially present in the metabarcoding data but removed during filtering due to low read counts. Metabarcoding of lysates retrieved only 3 to 4 reads of *Pemphredon* in a few samples, while metagenomics detected it in three samples with a classification range of 100,000 to 5 million unique minimisers. Notably, metabarcoding of homogenates recovered significantly higher read numbers for *Pemphredon*, exceeding the 100‐read threshold in all relevant samples. This suggests that the mild lysis protocol was less effective at extracting DNA from these wasps.

Among the 238 genus‐sample detections unique to metagenomics, 82.8% were detected by lysate‐based metabarcoding. Homogenate metabarcoding increased the percent to 96.2%. Six complete dropouts in homogenate metabarcoding appear to result from misidentifications in the metagenomics dataset. Three low‐read detections in the homogenate metabarcoding data are likely true mismatches caused by amplification bias.

Mild‐lysis metabarcoding detected 343 genus‐sample occurrences above the 100‐read threshold, of which 146 (34.5%) were not detected by metagenomics. However, lysate metabarcoding exhibited more detection failures, especially for heavily sclerotised insects, such as *Pemphredon* (digger wasps), *Formica* (ants), *Cantharis* (soldier beetles) and *Bombus* (bumble bees). These failures suggest that the mild lysis protocol released insufficient DNA for robust detection of such taxa.

Two apparent genus‐level mismatches, *Agriphila* and *Charanyca* (Lepidoptera), in the lysate metabarcoding data were traced to taxonomic annotation errors in the reference database (Iwaszkiewicz‐Eggebrecht et al. 2025). These mismatches underscore the challenges of accurate taxonomic assignment in metabarcoding workflows.

In conclusion, dropouts in lysate‐based metabarcoding were largely attributable to inadequate DNA release from heavily sclerotised or furry insects, rather than amplification bias. In contrast, homogenate‐based metabarcoding provided a more comprehensive recovery of genera, particularly for challenging taxa, and better alignment with metagenomic results.

## Appendix II Unique Minimisers Assigned to Common Bacterial Contaminants

Heatmap showing the number of unique minimisers per million reads across all samples and common bacterial contaminant genera (Salter et al. 2014). Bacterial genera are sorted by phylum on the *x* axis, and samples are shown as columns.
